# Impacts of Utensil Conditions on Consumer Perception and Acceptance of Food Samples Evaluated under In-Home Testing during the COVID-19 Pandemic

**DOI:** 10.3390/foods12050914

**Published:** 2023-02-21

**Authors:** Asmita Singh, Han-Seok Seo

**Affiliations:** Department of Food Science, University of Arkansas, 2650 North Young Avenue, Fayetteville, AR 72704, USA

**Keywords:** COVID-19, hedonic, in-home testing, sensory, utensil, fork, spoon, bowl

## Abstract

Sensory professionals are looking for alternative ways to conduct laboratory sensory testing, especially central location testing (CLT), during the COVID-19 pandemic. One way could be conducting CLTs at home (i.e., in-home testing). It is questionable whether food samples under in-home testing should be presented in uniform utensils, as it does so under laboratory sensory testing. This study aimed to determine whether utensil conditions could affect consumer perception and acceptance of food samples evaluated under in-home testing. Sixty-eight participants (40 females and 28 males) prepared chicken-flavored ramen noodle samples and evaluated them for attribute perception and acceptance, under two utensil conditions, using either their utensils (“Personal”) or uniform utensils provided (“Uniform”). Participants also rated their liking of forks/spoons, bowls, and eating environments, respectively, and attentiveness to sensory evaluation under each utensil condition. Results of the in-home testing showed that participants liked ramen noodle samples and their flavors under the “Personal” condition significantly more than under the “Uniform” condition. Ramen noodle samples evaluated under the “Uniform” condition were significantly higher in terms of saltiness than those evaluated under the “Personal” condition. Participants liked forks/spoons, bowls, and eating environments used under the “Personal” condition significantly more than those used under the “Uniform” condition. While overall likings of ramen noodle samples, evaluated under the “Personal” condition, significantly increased with an increase in hedonic ratings of forks/spoons or bowls, such significant correlations were not observed under the “Uniform” condition. In other words, providing uniform utensils (forks, spoons, and bowls) to participants in the in-home testing can reduce the influences of utensils on consumer likings of ramen noodle samples evaluated at home. In conclusion, this study suggests that sensory professionals should consider providing uniform utensils when they want to focus solely on consumer perception and acceptance of food samples by minimizing influences of environmental contexts, especially utensils, in the “in-home” testing.

## 1. Introduction

The Coronavirus Disease 2019 has caused a global pandemic, with variants causing frequent rises in cases for more than two years [1]. Despite worldwide vaccination attempts, there have been over 651 million confirmed cases and 6.6 million confirmed deaths as of 23 December 2022 [2]. Over this period, professionals around the world have experienced changes in their workplace, and remote work became popular to prevent the further spread of the disease. Moreover, pandemic preparedness is being given greater importance to prevent the losses caused during the current and potential episodes. This includes creating plans to not only avoid the spread of diseases, but to also maintain essential work and the economy as we do so.

The sensory and consumer science field is no stranger to working disruptions caused by the pandemic, as many methodologies, including central location testing, descriptive sensory analysis, and focus group interviews, were not feasible with safety guidelines. Since these procedures often require gatherings of individuals in controlled environments, sensory professionals have had to find alternate ways to conduct testing, as social distancing became a commonly used intervention during the current pandemic period [3,4]. Some methodologies, like focus group interviews and one-on-one interviews, were easily modified using video conferencing platforms. However, these methodologies are restricted in their use; for example, where controlled test conditions are required, temperature-sensitive and time-sensitive samples are tested, and elaborate sample preparation is needed. Descriptive sensory analysis using remote platforms was also common, as scientists found ways to conduct testing in a safe home environment [5,6]. An interesting methodology to emerge during the pandemic was sensory testing in vehicles, which could be used when samples could not be sent home to participants due to issues with confidentiality, preparation, or presentation limitations [3]. Their “drive-in booth” condition asked participants to hook a plastic tray onto the steering wheel, where they were served samples. This condition did not differ from a laboratory sensory booth condition, with respect to sensory, hedonic, and emotional responses to beverage samples. It made participants feel safer, and they felt it was more consistent with their real-world experience. However, the drive-in booth condition might be influenced by the weather, and it might have a limitation in testing some types of test samples (e.g., those with strong odors). Dinnella et al. [5] performed remote testing using online platforms and concluded that discrimination tests, descriptive analysis, temporal dominance of sensation (TDS) test, and check-all-that-apply (CATA) tests can be conducted remotely, with similar results to a laboratory setting’s results. In their study, comparisons were made between laboratory sensory testing and remote sensory testing at home. For the remote home testing session, each participant received a box of all the materials required for testing and set them up themselves. Then, they joined a video call where a session leader provided instructions to begin the study. Similar results were seen between the laboratory and home environment for the tests, except for the tetrad test. They mentioned that odorants in the testing environment may have caused this difference in the tetrad test. This emphasizes that potential sources of variability and bias (e.g., environmental contexts) should not be introduced in analytic types of sensory testing.

**Figure 1:**
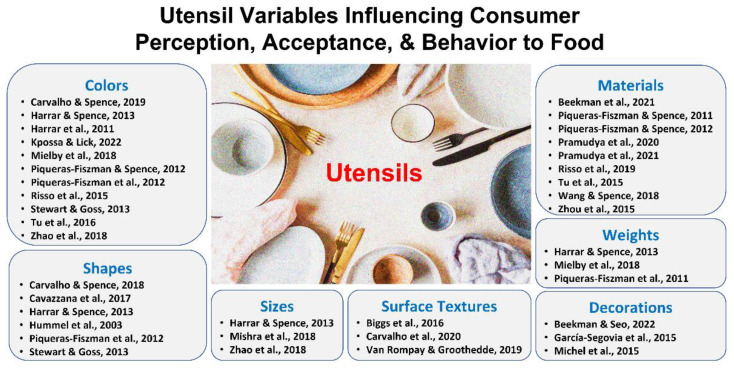
Summary of previous studies regarding the effects of utensil variables on consumer perception, acceptance, and behavior to food: colors [7,8,9,10,11,12,13,14,15,16,17], shapes [8,13,15,18,19,20], sizes [8,17,21], surface textures [22,23,24], materials [25,26,27,28,29,30,31,32,33], weights [8,11,34], and decorations [35,36,37]. The image of utensils was adapted with permission from ref. [42]. 2023, Olga Peshkova, Dreamstime.com, accessed on 19 February 2023.

Reducing the impacts of the environmental contexts on sample evaluation should be an important step to ensure the reproducibility and validity of the results obtained from in-home testing. Previous studies have found that consumer perception and acceptance of food or beverage samples change with environmental contexts, ranging from macro-level variables (e.g., geographical location, eating place, or climate) to micro-level variables (e.g., table setting, cutlery items, ambient lighting, or background sound) [7,8,9,10,11,12,13,14,15,16,17,18,19,20,21,22,23,24,25,26,27,28,29,30,31,32,33,34,35,36,37,38,39,40,41]. Notably, as shown in Figure 1, previous studies have demonstrated variations in consumer perception or acceptance of food or beverage samples as functions of utensil factors, such as colors [7,8,9,10,11,12,13,14,15,16,17], shapes [8,13,15,18,19,20], sizes [8,17,21], surface textures [22,23,24], materials [25,26,27,28,29,30,31,32,33], weights [8,11,34], and decorations [35,36,37]. For example, Tu et al. [16] demonstrated that food served on a red plate was perceived as spicier than that served on a white or green plate. Carvalho and Spence [7] also found that congruency of cup colors with the type of specialty coffee can increase liking and decrease the perceived acidity of the coffee beverage consumed. In addition to the color cues, the weights or sizes of containers have been found to affect consumer perception of food or beverage samples [8,11,17,34]. Yogurt consumed in heavier bowls was significantly more liked, perceived to be denser, and of a higher cost compared to those consumed in lighter bowls [34]. Interestingly, yogurt was also perceived as denser and more expensive when tasted with a lighter plastic spoon, rather than with heavier spoons [8]. Food consumed with heavier, banquet cutlery items was liked more, and rated as being more artistic and expensive than food consumed with lighter cutlery items [37]. Finally, the materials of cutlery or the food container have been found to significantly influence consumer perception of food samples [25,26,27,28,29,30,31,32,33]. Spoon materials affected the perceived quality and liking of yogurt [26] and cream [27]. Tu et al. [31] also found that cold tea contained in glass cups was perceived to be sweeter and colder than cold tea contained in plastic cups. Recently, studies also showed the influence of straw materials [25,29]. The taste of tea, with copper and stainless-steel straws, was liked more for its flavor, mouthfeel, and overall impression [29]. Interestingly, the material influence can also be seen for utensil components that do not directly touch the food itself. Pramudya et al. [28] found that cup sleeve materials can influence the taste perception of hot coffee, while Wang and Spence [32] found a similar effect of touching a swatch of velvet or sandpaper while simultaneously drinking wine on the aroma of wine. These examples show how participants’ home utensils could bring overall variations in the results, which can be especially detrimental to reformulation studies.

The ultimate goal of this study is to develop an effective methodology of in-home testing that can be an alternative to a laboratory sensory testing, especially during a current or potential pandemic. As an initial step to achieve the goal, this study aimed to test whether sensory perception and liking of food samples could differ as a function of utensil variables by comparing consumer responses to the same ramen noodles between two different utensil conditions: one using their personal utensils and the other using a laboratory provided, white colored plastic bowl, spoon, and fork. In this study, we hypothesized that consumer perception and acceptance of food samples evaluated at home would differ between two utensil conditions, highlighting the need to consider utensil variables when conducting a laboratory sensory testing at home during epidemic or pandemic periods.

## 2. Materials and Methods

### 2.1. Participants

A total of 71 participants (43 females and 28 males), aged from 18 to 65 years old, were recruited using the consumer profile database of the University of Arkansas Sensory Science Center (Fayetteville, AR, USA). Three participants were excluded from the data analysis as they did not complete the study, leaving a final sample of 68 participants (40 females and 28 males), with a mean age of 38 years [standard deviation (SD) = 11 years]. Table 1 shows demographic profiles of the participants. The participants self-reported as being ramen noodle consumers (at least once every 2–3 months) and having no major diseases or conditions that would affect their taste or smell sensitivities. They also self-reported as being likers of chicken flavored ramen noodles. Additionally, since this was a test conducted at home, only participants who could cook ramen noodles at their home were selected.

### 2.2. Food Sample Kits and Utensil Conditions

Chicken flavored ramen noodles (Top Ramen, Nissin Foods Co. Inc., Gardena, CA, USA) were wrapped in white paper to conceal brand information. The same noodles were used for both conditions of the study; however, they were labeled with two separate three-digit codes (i.e., “190” and “213”), making two different samples. Participants were asked to taste and evaluate chicken flavored ramen noodles under two different utensil conditions: (1) personal utensil (“Personal”), where participants were asked to use their own utensils, and (2) uniform utensil (“Uniform”), where participants were asked to use the uniform utensils we provided.

Each participant received two sample kits for the “Personal” and “Uniform” conditions. A sample kit for “Personal” or “Uniform” conditions included the chicken flavored ramen noodle identified with a three-digit code, cooking directions, instructions for sensory evaluation, and the access link and code of the remote platform. A sample kit for the “Uniform” condition also contained a 148 mL white plastic water cup (Dart Container Corporation, Mason, MI, USA), a white paper napkin (Essity Professional Hygiene North America LLC, Philadelphia, PA, USA), a white plastic spoon (Walmart Inc., Bentonville, AR, USA), a white plastic fork (Walmart Inc., Bentonville, AR, USA), and a 532 mL white plastic bowl (KCH Corporation, Brooklyn, NY, USA). The cooking directions were adapted from the noodle packet itself and contained additional instructions to ensure uniform cooking.

### 2.3. Procedure

The protocol of this study (#2009287230) was approved by the Institutional Review Board of the University of Arkansas (Fayetteville, AR, USA). Prior to participation, the experimental procedure was fully explained to each participant, and a written informed consent was obtained from each participant.

Participants were asked to pick up sample packets one week apart and complete each testing at home within 24 h of pick up. Since test samples were shelf stable, storage instructions were not specified. Participants were asked to refrain from smoking, eating, or drinking anything (except for water) for at least 2 h prior to sample evaluation [43]. They were asked to conduct the test alone in a quiet place, with no distractions (e.g., using cell phones, watching TV, playing games, and listening to music, etc.), at their houses. Participants were asked to start the test by logging into the data collection platform. The platform then provided detailed instructions for the test procedure, including cooking directions (for details, see Supplementary Material). Prior to cooking, participants were asked to rate their hunger/fullness and feeling on 9-point category scales ranging from 1 (“extremely hungry” or “extremely bad”) to 9 (“extremely full” or “extremely good”), respectively. In other words, this experimental setting was designed to ask participants to prepare and evaluate ramen noodle samples twice (i.e., “Personal” and “Uniform”) in the same conditions, except for the utensil-related variables, thereby allowing that the utensil condition is the only variable influencing participants’ perception and liking of ramen noodle samples.

After preparing the ramen noodle sample based on the cooking directions, they were asked to pour the noodles and broth in either their own bowls (“Personal” condition) or the uniform bowls provided (“Uniform” condition) and then wait for instructions shown on the remote platform to begin consuming the sample. The two utensil conditions (“Personal” and “Uniform”) were randomized among participants so that each condition was presented first an equal number of times. To ensure that participants followed the provided instructions on cooking, they were asked to specify cooking medium and additional ingredients used during cooking. As shown in cooking directions of the Supplementary Material, participants were asked to cook ramen noodles only using seasonings from the flavor packet provided. Note: participants were asked not to add any other ingredients into the noodles and broth. After completing the cooking process, participants were asked to rate hunger/fullness and feeling again on the 9-point category scales (see above).

Participants were asked to evaluate the noodles with respect to both intrinsic and extrinsic factors. After first tasting the sample (i.e., the first bite), participants rated hedonic impressions of the noodles in terms of (1) overall liking, (2) overall flavor liking, (3) broth texture liking, and (4) noodle texture liking on 9-point hedonic scales, ranging from 1 (“dislike extremely”) to 9 (“like extremely”). Participants also rated their willingness to eat again on a 9-point category scale, ranging from 1 (“extremely unwanted”) to 9 (“extremely wanted”). They also rated attribute intensities of the noodles with respect to (1) overall flavor, (2) chicken flavor, and (3) saltiness on 9-point category scales, ranging from 1 (“extremely weak”) to 9 (“extremely strong”). Thereafter, participants were asked to eat the noodles as much as they wanted (hereafter referred to as “last bite”) and rate their overall liking and willingness to eat again, as they did above. To ensure that participants consumed test samples during sample evaluation, they were asked to specify the amount of test samples they ate among the five options: <25%, 25%, 50%, 75%, and 100%.

Participants were asked to describe the colors and materials of forks/spoons and bowls used in the sample evaluation and rate their likings of (1) forks/spoons, (2) bowls, and (3) eating environments under either utensil condition on 9-point hedonic scales, ranging from 1 (“dislike extremely”) to 9 (“like extremely”). Lastly, participants rated their attentiveness to sample evaluation, in either utensil condition, on a 9-point scale, ranging from 1 (“extremely distracting) to 9 (“extremely attentive”). Supplementary Material shows details of instructions and scales used in this study.

### 2.4. Data Analysis

Data analysis was performed using SPSS 28.0 for Windows^TM^ (IBM SPSS Inc., Chicago, IL, USA) and XLSTAT statistical software (Addinsoft, New York, NY, USA). The Shapiro–Wilk test identified that the null hypothesis, stating the data came from a normally distributed population, was rejected for ratings of individual variables measured in this study (*p* < 0.05). The transformation process of non-normally distributed data into normally distributed data was not conducted because transformed variables may lose their identity [44]. Therefore, because of the current experimental design (e.g., a small sample size and type of scales used in this study) and non-normality of data distribution, non-parametric methods were used for data analysis in this study (see also [45]).

A Wilcoxon signed-rank test was conducted to compare the two utensil conditions, with respect to participants’ hungers and feelings, measured either before or after sensory evaluation. To test whether the two utensil conditions differed in terms of (1) hedonic impressions (i.e., overall liking after the first bite, overall flavor liking, noodle texture liking, broth texture liking, and overall liking after the last bite), (2) attribute intensities (overall flavor, chicken flavor, and saltiness), (3) willingness to eat again (WTEA) (WTEA after the first bite and WTEA after the last bite), or (4) extrinsic cues (fork/spoon liking, bowl liking, eating environment liking, and attentiveness to sensory evaluation), Wilcoxon signed-rank tests were performed. Spearman’s correlation analyses were performed to determine how ratings of overall liking for the ramen noodle could be associated with the ratings related to extrinsic cues (fork/spoon liking, bowl liking, eating environment liking, and attentiveness to sensory evaluation). To visualize associations between overall liking of ramen noodle and likings of fork/spoon or bowl, multiple correspondence analysis (MCA) was conducted. For MCA, 9-point ratings of the four variables (overall liking of ramen noodle, fork/spoon liking, bowl liking, and environment liking) were categorized into three groups: low (1 to 3), medium (4 to 6), and high (7 to 9). A chi-squared test was used to determine a difference between the two utensil conditions in terms of the proportion of sample consumption. A statistically significant difference was defined to be present when *p* < 0.05.

## 3. Results

### 3.1. Comparisons between Utensil Conditions in Terms of Hunger/Fullness or Feeling

A Wilcoxon signed-rank test revealed no significant difference between the two utensil conditions, with respect to hunger/fullness (*Z* = −0.07, *p* = 0.95) or feeling (*Z* = −1.26, *p* = 0.21) measured before sample evaluations. This result suggests that the impact of hunger and feeling were minimal when we investigated the effects of utensil conditions on consumer perception and liking of ramen noodle samples. Additionally, there were no significant differences between the two utensil conditions in terms of hunger/fullness (*Z* = −0.25, *p* = 0.80) or feeling (*Z* = −1.47, *p* = 0.14), measured at the end of sample preparation, suggesting that utensil conditions did not affect participants’ hunger/fullness and feeling during their sample preparation. Therefore, variables of hunger/fullness and feeling were not considered for subsequent statistical analyses.

Under the “Personal” condition, 68 participants self-reported that they ate 100% (*n* = 29, 42.6%), 75% (*n* = 19, 27.9%), 50% (*n* = 13, 19.1%), 25% (*n* = 6, 8.8%), and <25% (*n* = 1, 1.5%). Similarly, under the “Uniform” condition, the participants ate 100% (*n* = 30, 44.1%), 75% (*n* = 16, 23.5%), 50% (*n* = 19, 27.9%), 25% (*n* = 3, 4.4%), and <25% (*n* = 0, 0.0%). A chi-squared test revealed no significant difference between the two utensil conditions, with respect to the proportion of sample consumption (*χ*^2^ = 3.40, *p* = 0.49), suggesting that utensil conditions did not affect the amount of consumption during sample evaluation.

### 3.2. Comparisons between Utensil Conditions in Terms of Consumer Perceptions and Likings of Ramen Noodle Samples

**Figure 2:**
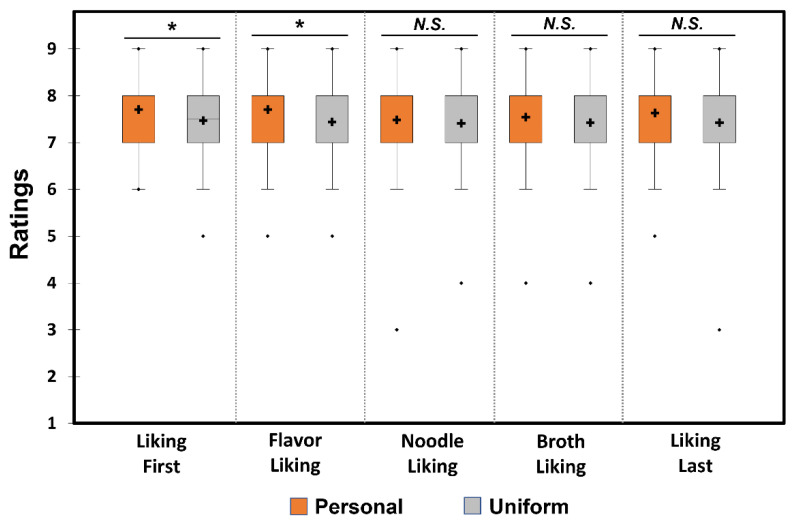
Comparisons between the two utensil conditions, “Personal” and “Uniform”, with respect to hedonic ratings of ramen noodle samples: overall liking after the first bite (“Liking First”), flavor liking, noodle liking, broth liking, and overall liking after the last bite (“Liking Last”). In each box plot, the cross and the central horizontal bar represent the mean and the median, respectively. The lower and upper limits of each box indicate the first and third quartiles, respectively. Points below or above the lower or upper bounds indicate outliers. * indicates a significant difference determined by Wilcoxon signed-rank test at *p* < 0.05. *N.S.* indicates no significant difference at *p* < 0.05.

A Wilcoxon signed-rank test revealed a significant difference between the two utensil conditions (“Personal” and “Uniform”), with respect to overall liking (*Z* = −2.24, *p* = 0.03) or overall flavor (*Z* = −2.06, *p* = 0.04) of ramen noodle samples evaluated after the first bite. As shown in Figure 2, participants liked ramen noodle samples and their flavors under the “Personal” condition significantly more than those under the “Uniform” condition. However, there were no significant differences between the two utensil conditions in terms of texture liking of ramen noodle (*Z* = −0.44, *p* = 0.66) or broth (*Z* = −0.88, *p* = 0.38) evaluated after the first bite. No significant difference between the two utensil conditions was observed in the overall liking of ramen noodle samples evaluated after the last bite (i.e., at the end of consumption) (*Z* = −1.30, *p* = 0.19).

**Figure 3:**
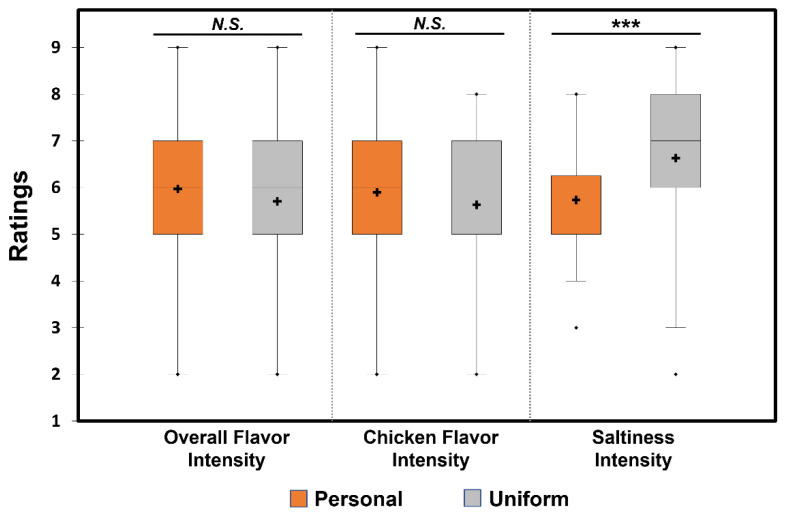
Comparisons between the two utensil conditions, “Personal” and “Uniform”, with respect to attribute intensity: overall flavor, chicken flavor, and saltiness. In each box plot, the cross and the central horizontal bar represent the mean and the median, respectively. The lower and upper limits of each box indicate the first and third quartiles, respectively. Points below or above the lower or upper bounds indicate outliers. *** indicates a significant difference determined by Wilcoxon signed-rank test at *p* < 0.001. *N.S.* indicates no significant difference at *p* < 0.05.

Figure 3 shows comparisons between the two utensil conditions, with respect to attribute intensity (i.e., overall flavor, chicken flavor, and saltiness). No significant differences between the two utensil conditions were observed in the intensity ratings of overall flavor (*Z* = −1.60, *p* = 0.11) and chicken flavor (*Z* = −1.70, *p* = 0.09). Participants rated the ramen noodle samples evaluated under the “Uniform” condition significantly saltier than those evaluated under the “Personal” condition (*Z* = −3.65, *p* < 0.001).

### 3.3. Comparisons between Utensil Conditions in Terms of Willingness to Eat Ramen Noodle Samples

A Wilcoxon signed-rank test found that two utensil conditions did not differ, with respect to WTEA rated after the first bite (*Z* = −1.77, *p* = 0.08) or the last bite (*Z* = −1.71, *p* = 0.09) (Table 2).

### 3.4. Comparisons between Utensil Conditions in Terms of Consumer Perceptions and Likings of Extrinsic Cues

While participants used white plastic forks/spoons and white plastic bowls when consuming ramen noodle samples in the “Uniform” utensil condition, they used diverse forks/spoons and bowls in terms of color and material in the “Personal” condition (Table 3). Specifically, in the “Personal” utensil condition, 57 participants (83.8%) ate ramen noodle samples using silver metal forks/spoons. More than half of participants used white (*n* = 27, 42.8%) or multi-colored (*n* = 17, 25.0%) bowls. Additionally, more than half of participants ate ramen noodles contained in ceramic (*n* = 40, 58.8%) or plastic (*n* = 17, 25.0%) bowls.

**Figure 4:**
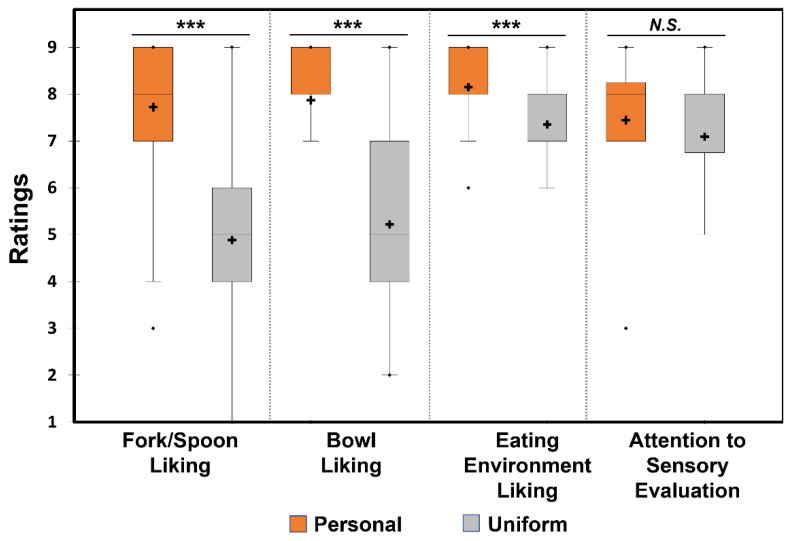
Comparisons between the two utensil conditions, “Personal” and “Uniform”, with respect to extrinsic cues: fork/spoon liking, bowl liking, eating environment liking, and attention to sensory evaluation. In each box plot, the cross and the central horizontal bar represent the mean and the median, respectively. The lower and upper limits of each box indicate the first and third quartiles, respectively. Points below or above the lower or upper bounds indicate outliers. *** indicates a significant difference determined by Wilcoxon signed-rank test at *p* < 0.001. *N.S.* indicates no significant difference at *p* < 0.05.

A Wilcoxon signed-rank test revealed a significant difference between the two utensil conditions, with respect to fork/spoon liking (*Z* = −6.30, *p* < 0.001), bowl liking (*Z* = −6.35, *p* < 0.001), or eating environment liking (*Z* = −4.16, *p* < 0.001), with higher likings under the “Personal” condition (Figure 4). There was no significant difference between the two utensil conditions in terms of attention to sensory evaluation (*Z* = −1.83, *p* = 0.07).

### 3.5. Associations between Consumer Likings of Extrinsic Cues and Ramen Noodle Samples Consumed under Each Utensil Condition

Table 4 represents Spearman’s correlation coefficients, with respect to hedonic ratings of ramen noodle samples and individual extrinsic cues (forks, bowls, and eating environments) as a function of utensil condition. In the “Personal” condition, overall likings of the ramen noodle sample, evaluated after the last bite, were found to be positively correlated with likings of forks/spoons (*rho*_68_ = 0.31, *p* = 0.01), bowls (*rho*_68_ = 0.42, *p* < 0.001), or eating environments (*rho*_68_ = 0.31, *p* = 0.01). In other words, the more participants liked the forks/spoons, bowls, and eating environments they used to eat ramen noodle samples, the more they liked the ramen samples. This association was only found for the eating environment liking in the “Uniform” condition, where overall likings of the ramen noodle sample, evaluated after the last bite, were found to be correlated with likings of eating environments (*rho*_68_ = 0.32, *p* = 0.009). Significant correlations were not observed with likings of forks/spoons (*rho*_68_ = 0.23, *p* = 0.06) or bowls (*rho*_68_ = 0.13, *p* = 0.31). Overall likings of the ramen noodle sample, evaluated after the last bite, were not found to be correlated with ratings of attention to sensory evaluation under either “Personal” (*rho*_68_ = 0.12, *p* = 0.33) or “Uniform” conditions (*rho*_68_ = −0.01, *p* = 0.92).

**Figure 5:**
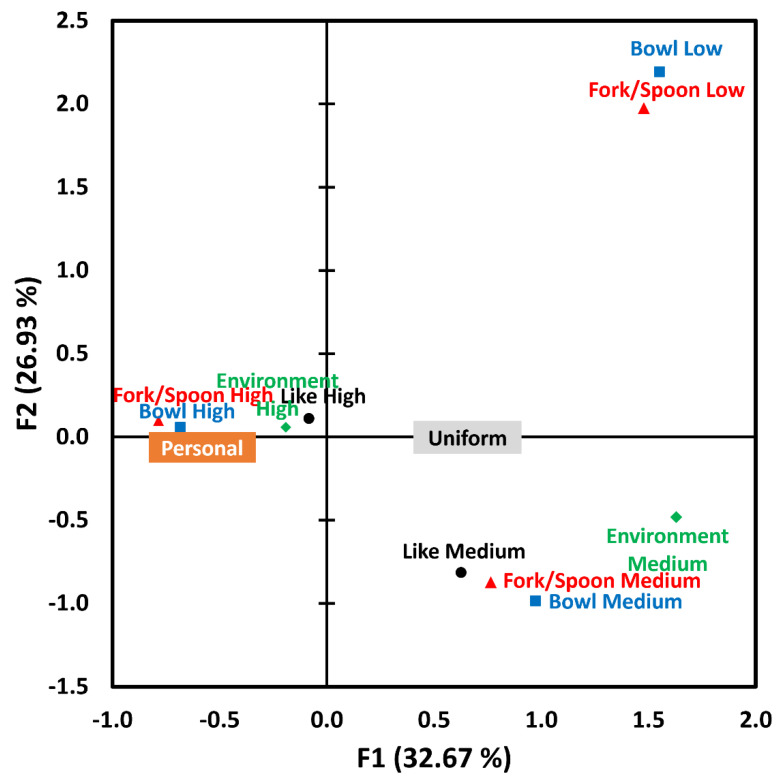
A biplot of multiple correspondence analysis (MCA) among four sub-groups of likings for ramen noodle samples (black circle), forks/spoons (red triangle), bowls (blue square), or eating environments (green diamond). Because of none (for “Personal” condition) or a low frequency of response (*n* = 2 for “Uniform” condition), the low group of liking for ramen noodle samples was not included. Additionally, the low group of liking for eating environments (*n* = 1 for “Uniform” condition) was not included for MCA. Utensil conditions, “Personal” and “Uniform”, were used as a supplementary variable.

Figure 5 represents a biplot of MCA among four sub-groups of likings for ramen noodle samples (black circle), forks/spoons (red triangle), bowls (blue square), or environments (green diamond), accounting for 59.60% of total variance. Because of none (for “Personal” condition) or a low frequency of response (*n* = 2 for “Uniform” condition), the low group of liking for ramen noodle samples was not included. In the same manner, the low group of liking for eating environments (*n* = 1 for “Uniform” condition) was excluded for MCA. As shown in Figure 5, medium (or high) groups of likings for ramen noodle samples, fork/spoons, bowls, or environments were closely placed on the biplot. Additionally, high groups of likings for ramen noodle samples, forks/spoons, bowls, or environments were closely associated with the “Personal” condition, employed as a supplementary variable, which supports the above results (Figure 2 and Figure 4).

## 4. Discussion

### 4.1. Influence of Utensil Conditions on Consumer Perceptions and Likings of Ramen Noodle Samples

We found that the overall liking on first impression and overall flavor liking was significantly higher for the noodles when consumed with personal utensils. Since personal utensils could vary in weight, size, material, and shape among the participants, these property variations could influence likings of ramen noodle samples [7,8,9,10,11,12,13,14,15,16,17,18,19,20,21,22,23,24,25,26,27,28,29,30,31,32,33,34,35,36,37]. This observation can be explained by the halo effect, i.e., the ramen noodles might be rated more positively because the utensils used to eat them were very well-liked [45,46]. Nevertheless, the higher overall liking rating for the “Personal” condition was not seen during the last bite (Figure 2). Previous studies have shown that, when consumers are familiar with a product, the relative importance of the product extrinsic cues on consumers’ evaluations reduces [47,48]. Because the participants in this study were habitual consumers and likers of ramen noodles, the utensil conditions-induced difference in overall liking of the ramen noodles might be decreased when evaluated at the end of their consumption.

The saltiness intensity of noodles consumed in the “Uniform” condition was rated to be significantly higher than that consumed in the “Personal” condition (Figure 3). Harrar et al. [9] had found effects of the color of the bowl on popcorn and theorized that the color of the bowl could influence mood and emotions, and color-taste associations could influence sensory perceptions. Other studies have also shown that the white color of the plates increased the intensity of the dominant taste, and the flavor intensity of the food itself [13,15], although the test samples in these studies were desserts, with the dominant taste being sweet. Since previous studies have shown that white color is often associated with saltiness [49,50], the presence of the white bowls in our study could have enhanced the dominant salty taste of ramen noodles. Another plausible explanation is related to a difference between the two utensil conditions, with respect to bowl material. As listed in Table 3, 58.8% and 25.0% of participants used ceramic and plastic bowls, respectively, in the “Personal” condition. When comparing ratings of saltiness intensity within the “Personal” condition, a Mann–Whitney test revealed that participants rated ramen noodle samples served in plastic bowls (mean = 6.00, median = 6.00) significantly saltier than those served in ceramic bowls (mean = 5.75, median = 5.50) (*p* = 0.01), supporting the result that the “Uniform” condition using plastic bowls exhibited higher saltiness than the “Personal” condition, in which 58.8% of participants used ceramic bowls (Figure 3). The difference between plastic and ceramic bowls, with respect to saltiness of ramen noodle samples within the “Personal” condition, might be related to the broth temperature-induced saltiness perception [51,52] because thermal conductivity of the two materials may be different. However, since temperatures of the ramen noodle samples were not measured in this study, a further study should be conducted to confirm this assumption.

### 4.2. Consumer Perceptions and Likings of Extrinsic Cues and Their Influences on Sensory and Hedonic Ratings of Ramen Noodle Samples

Participants rated their liking for forks/spoons, bowls, and environments under the “Personal” condition significantly higher than under the “Uniform” condition. These results were expected since participants would be more familiar and comfortable with their own utensils, which might have emotional significance to them, compared to plastic utensils provided by researchers. Previous studies have shown that familiarity and repeated exposure influence likings of test samples [53,54,55]. Thus, familiarity and liking of utensils, and the unconscious positive emotions elicited from them, might have contributed to increased likings of eating environments in the “Personal” condition [56], which is clearly observed in Figure 5. In other words, higher likings of ramen noodles, forks/spoons, bowls, and eating environments were closely placed with the “Personal” condition.

The ratings of overall liking of the ramen noodle samples, evaluated after the last bite, were positively correlated with likings of forks/spoons, bowls, or eating environments in the “Personal” condition. As previously discussed, this indicates the presence of the halo effect, where the utensil liking positively influenced the liking of the noodles itself [45,46]. Another phenomenon that could explain this effect is the haptic transference of perception, as demonstrated by previous studies, where hand-feel touch stimuli of the receptacle can influence the pleasantness [30] and sensory ratings [22,28,31] of the food or beverage itself. This phenomenon was demonstrated by a study with Asian noodles where presenting the noodles in a ceramic receptacle resulted in higher pleasantness and wanting scores than when presented in a stainless-steel receptacle [33]. Given that the personal utensils used by participants were of better quality than the ones provided in the “Uniform” condition, the pleasantness of the receptacle and utensils could have transferred into the pleasantness ratings of the noodle samples. However, in the “Uniform” condition, overall liking of the ramen noodle samples, evaluated after the last bite, correlated with the eating environment liking, but not with forks/spoons or bowl liking. Since the utensils in the “Uniform” condition can be perceived as being neutral, these results show that they can be good for sensory testing, as the hedonic impression of the food samples are unaffected by the liking of utensils. Notably, overall likings of the ramen noodle samples were not correlated with ratings of attention to sensory evaluation under either condition. This result shows that participants did not feel a difference in their level of attention, although there were subconscious influences of utensil conditions on their ratings.

### 4.3. Implications and Limitations

This study tested a methodology, using “in-home booth”, where participants create a laboratory booth-like environment at home, using uniform utensils. A similar study compared whether repeated exposure to food samples at home versus lab would influence consumer perception and ratings [57]. They found that the explicit ratings of liking and attribute intensity of foods tested repeatedly at home, and in the lab, did not show differences between test locations. It should be noted that the differences between their home and lab conditions were smaller than a regular home-use study, since participants consumed the same quantity of the same test foods, in the same containers, at similar times in isolation. This implies that in-home booth testing can potentially produce similar results as in the laboratory. The in-home booth testing provides advantages over alternative sensory testing methods, such that they mimic a laboratory scenario by including uniformity in the preparation and evaluation methods. As was observed in this study, complicated preparation procedures can be successfully done by general consumers, if clear and detailed instructions are provided [58]. This key element differentiates it from the traditional home-use testing method, where product evaluation in natural environments is required. In addition to the drive in-booth test [3], in-home booth testing using uniform utensils can be used for samples that require kitchen preparation and can be temperature sensitive.

A limitation of this study is that only one product was used as a test sample. To generalize the effect of uniform utensils, a further study is needed to conduct consumer acceptance testing with multiple samples. It would also be interesting to test whether providing uniform utensils can be superior to allowing personal utensils, in terms of discriminability among multiple samples within a session, and reproducibility across repeated tests. It should also be determined whether participants’ perception and liking of the test samples, evaluated in the laboratory sensory booths, can be better matched to those in the “Uniform” utensil condition than those in the “Personal” condition. Moreover, although some contextual variables (e.g., absence of other diners, quiet place, no distractions, etc.) were controlled, impacts of other influential factors (e.g., time of sensory evaluation) should be investigated in future studies. While participants were asked to test ramen noodle samples twice (i.e., “Personal” and “Uniform”) in the same places, there might have been unnoticed or overlooked differences between the two sessions, with respect to eating environments (e.g., changes in furniture, light color, or ambient temperature) [40,59,60,61]. Therefore, we should carefully consider the possibility that other environmental factors, in addition to the utensil condition, might have been different between the two utensil conditions. Lastly, studies with other populations (e.g., children, the elderly, or individuals with reduced sensory functions) could help sensory professionals better understand whether the “in-home testing” under the “Uniform” utensil condition can be applied in a wide variety of sensory testing.

## 5. Conclusions

As the COVID-19 pandemic continues to remain a health and safety concern with the threat of new variants, it is imperative to keep alternate sensory testing methods handy so that product development and sensory evaluation can continue. Since laboratory sensory testing (especially central location testing) is not feasible in times of a lockdown or social distancing guidelines, we considered an alternative method of sensory testing, i.e., “in-home testing”. In this method, participants were provided with uniform utensils, along with guidelines on preparing the sample. We found that sensory ratings for the noodle samples varied with the type of utensil being used. Specifically, saltiness intensity was rated higher when consumed from a white plastic bowl compared to other utensils. In the “Personal” utensil condition, overall liking of the ramen noodle positively correlated with likings of the forks/spoons and bowls, but such a relationship was not observed in the “Uniform” condition. In conclusion, uniform utensil usage is suggested in the in-home testing scenario to reduce the influence of extrinsic factors on the sensory characteristics of test samples.

## Figures and Tables

**Table 1:** Demographic profiles of the participants of this study.

Category	Subcategory	Frequency	Percentage (%)
Gender	Female	40	58.8
	Male	28	41.2
Age group	20 to 29 years old	22	32.4
	30 to 39 years old	20	29.4
	40 to 49 years old	12	17.6
	50 to 59 years old	13	19.1
	60 to 69 years old	1	1.5
Ethnicity	White/Caucasian	46	67.6
	Black/African-American	8	11.8
	Asian	7	10.3
	Hispanic	6	8.8
	Other	1	1.5
Annual household income	<$20,000	10	14.7
	$20,000–$39,999	19	27.9
	$40,000–$59,999	24	35.3
	$60,000–$79,999	6	8.8
	$80,000–$99,999	9	13.2
Education level	High school	4	5.9
	Associate degree	6	8.8
	Some college	11	16.2
	Bachelor’s degree	29	42.6
	Post graduate degree	18	26.5

**Table 2:** Comparisons between utensil conditions in terms of willingness to eat ramen noodle samples again (WTEA), rated after the first or the last bite.

	Utensil Condition		*Z*-Value (*p*-Value)
	Personal	Uniform	
WTEA after the first bite	7.32 ± 1.23 (8) ^1^	7.12 ± 1.18 (7)	−1.77 (0.08)
WTEA after the last bite	7.28 ± 1.36 (8)	6.99 ± 1.28 (7)	−1.71 (0.09)

^1^ Mean ± standard deviation (median).

**Table 3:** Colors and materials of the forks/spoons or bowls used by 68 participants in the two utensil conditions, “Personal” and “Utensil”.

Category	Sub-Category	Utensil Conditions	Color or Material (Frequency)
Forks/Spoons	Color	Personal	Black (1), Red (1), Silver (57), Tan (1), White (7), Yellow (1)
		Uniform	White (68)
	Material	Personal	Metal (57), Metal & Plastic (1), Plastic (9), Wood (1)
		Uniform	Plastic (68)
Bowls	Color	Personal	Black (2), Blue (6), Brown (3), Cream (1), Gray (1), Multi-colors (17), Orange (1), Pink (1), Purple (1), Red (3), Silver (1), Transparent (4), White (27)
		Uniform	White (68)
	Material	Personal	Ceramic (40), Glass (8), Metal (2), Plastic (17), Styrofoam (1)
		Uniform	Plastic (68)

**Table 4:** Associations of hedonic ratings of ramen noodle samples, evaluated after the last bite, with either hedonic ratings of individual extrinsic cues (forks/spoons, bowls, and eating environments) or ratings of attentiveness to sensory evaluation. (*n* = 68).

	Utensil Condition	
	Personal	Uniform
Likings of forks/spoons	0.31 (0.01) ^1^	0.23 (0.06)
Likings of bowls	0.42 (<0.001)	0.13 (0.31)
Likings of eating environments	0.31 (0.01)	0.32 (0.009)
Ratings of attention to sensory evaluation	0.12 (0.33)	−0.01 (0.92)

^1^ Spearman’s correlation coefficient (*p*-value).

## Data Availability

The data are not publicly available due to the Institutional Review Board protocol guideline.

## Supplementary Materials

The following supporting information can be downloaded at: https://www.mdpi.com/article/10.3390/foods12050914/s1, supplementary materials file.
