# Supplementary material for: Impacts of Utensil Conditions on Consumer Perception and Acceptance of Food Samples Evaluated under In-Home Testing during the COVID-19 Pandemic
Source: Foods. 2023 Feb 21;12(5):914. doi: 10.3390/foods12050914 (PMC10001261; doi:10.3390/foods12050914)
Supplement: Supplementary file 1 [file foods-12-00914-s001.zip › foods-2183237-supplementary.pdf]

Instruction prior to participating in the study:

Note: We ask that you refrain from smoking, eating, and drinking for 2 hours prior to your participation; drinking water is allowed. Did you refrain from smoking, eating, and drinking (except water) for 2 hours? If not yet, please try it later. Please note that you are required to cook and evaluate the ramen noodles alone, and away from any distractions. If you are ready to begin cooking, please press the button below.

Cooking directions:

1. Please practice caution while preparing this sample.
2. Boil 2 cups of water in a saucepan.
3. Add noodles, breaking up if desired.
4. Cook for 3 minutes (or until noodles are tender), stirring occasionally.
5. Remove from heat, add flavor packet (to the noodles and broth), stir well for 1 minute until flavoring is fully dissolved.
6. Please do not add any other ingredients into the noodles and broth.
7. Pour all the noodles and broth into a ~ 16 oz bowl of your liking. Because the noodles and broth are VERY HOT, please be careful.
8. PLEASE don't eat the sample yet. Click next once you are ready for evaluation.

Q1) Please select the cooking medium used for this study.

- □ Microwave
- □ Stove top
- □ Induction cooker
- □ Other

Q2) Please mention if ingredients other than noodle, water, and seasoning packet were used to prepare this sample. Please write N/A if nothing else was added.

Have you finished cooking the sample? If yes, please click "next".
If not, please finish cooking the sample and then press "next".

Questions and scales used in the study:

Hunger/Fullness

How HUNGRY or FULL do you feel now?

Extremely Hungry	Very Hungry	Moderately Hungry	Slightly Hungry	Neither Hungry nor Full	Slightly Full	Moderately Full	Very Full	Extremely Full
☐	☐	☐	☐	☐	☐	☐	☐	☐

Feeling

How GOOD or BAD do you feel now?

Extremely Bad	Very Bad	Moderately Bad	Slightly Bad	Neither Bad nor Good	Slightly Good	Moderately Good	Very Good	Extremely Good
☐	☐	☐	☐	☐	☐	☐	☐	☐

Hedonic impression

Please rate your impression of the OVERALL LIKING of the sample.

Dislike Extremely	Dislike Very Much	Dislike Moderately	Dislike Slightly	Neither Like nor Dislike	Like Slightly	Like Moderately	Like Very Much	Like Extremely
☐	☐	☐	☐	☐	☐	☐	☐	☐

Please rate your LIKING of OVERALL FLAVOR of the sample.

Dislike Extremely	Dislike Very Much	Dislike Moderately	Dislike Slightly	Neither Like nor Dislike	Like Slightly	Like Moderately	Like Very Much	Like Extremely
☐	☐	☐	☐	☐	☐	☐	☐	☐

Please rate your LIKING of NOODLE TEXTURE of the sample.

Dislike Extremely	Dislike Very Much	Dislike Moderately	Dislike Slightly	Neither Like nor Dislike	Like Slightly	Like Moderately	Like Very Much	Like Extremely
☐	☐	☐	☐	☐	☐	☐	☐	☐

Please rate your LIKING of BROTH TEXTURE of the sample.

Dislike Extremely	Dislike Very Much	Dislike Moderately	Dislike Slightly	Neither Like nor Dislike	Like Slightly	Like Moderately	Like Very Much	Like Extremely
☐	☐	☐	☐	☐	☐	☐	☐	☐

Please rate your OVERALL LIKING of the sample.

Dislike Extremely	Dislike Very Much	Dislike Moderately	Dislike Slightly	Neither Like nor Dislike	Like Slightly	Like Moderately	Like Very Much	Like Extremely
☐	☐	☐	☐	☐	☐	☐	☐	☐

Sensory attribute intensity

Please rate your impression of the INTENSITY of OVERALL FLAVOR of the sample.

Extremely Weak	Very Weak	Moderately Weak	Slightly Weak	Neither Weak nor Strong	Slightly Strong	Moderately Strong	Very Strong	Extremely Strong
☐	☐	☐	☐	☐	☐	☐	☐	☐

Please rate your impression of the INTENSITY of CHICKEN FLAVOR of the sample.

Extremely Weak	Very Weak	Moderately Weak	Slightly Weak	Neither Weak nor Strong	Slightly Strong	Moderately Strong	Very Strong	Extremely Strong
☐	☐	☐	☐	☐	☐	☐	☐	☐

Please rate your impression of the INTENSITY of SALTINESS of the sample.

Extremely Weak	Very Weak	Moderately Weak	Slightly Weak	Neither Weak nor Strong	Slightly Strong	Moderately Strong	Very Strong	Extremely Strong
☐	☐	☐	☐	☐	☐	☐	☐	☐

Want to eat again

Please rate how much you WANT to eat this sample again.

Extremely Unwanted	Very Unwanted	Moderately Unwanted	Slightly Unwanted	Neither Unwanted Nor Wanted	Slightly Wanted	Moderately Wanted	Very Wanted	Extremely Wanted
☐	☐	☐	☐	☐	☐	☐	☐	☐

Amount of sample consumption

Please select approximately the AMOUT of sample you ate.

☐ 100%

☐ 75%

☐ 50%

☐ 25%

☐ < 25%

Extrinsic factors

Please describe the COLOR of the FORK/SPOON you are using to evaluate this sample.

Please describe the MATERIAL (e.g., plastic, stainless steel, etc.) of the FORK/SPOON you are using to evaluate this sample.

Please rate your LIKING of the FORK/SPOON used in this study.

Dislike Extremely	Dislike Very Much	Dislike Moderately	Dislike Slightly	Neither Like nor Dislike	Like Slightly	Like Moderately	Like Very Much	Like Extremely
☐	☐	☐	☐	☐	☐	☐	☐	☐

Please describe the COLOR of the BOWL in which you served the ramen noodles for this evaluation.

Please describe the MATERIAL (e.g., plastic, stainless steel, etc.) of the BOWL in which you served the ramen noodles for this evaluation.

Please rate your LIKING of the BOWL used in this study.

Dislike Extremely	Dislike Very Much	Dislike Moderately	Dislike Slightly	Neither Like nor Dislike	Like Slightly	Like Moderately	Like Very Much	Like Extremely
☐	☐	☐	☐	☐	☐	☐	☐	☐

Please rate your LIKING of the EATING ENVIRONMENT of the evaluation of this sample.

Dislike Extremely	Dislike Very Much	Dislike Moderately	Dislike Slightly	Neither Like nor Dislike	Like Slightly	Like Moderately	Like Very Much	Like Extremely
☐	☐	☐	☐	☐	☐	☐	☐	☐

Please rate your DISTRACTING or ATTENTIVE was this environment for the evaluation of this sample.

Extremely Distracting	Very Distracting	Moderately Distracting	Slightly Distracting	Neither Distracting Nor Attentive	Slightly Attentive	Moderately Attentive	Very Attentive	Extremely Attentive
☐	☐	☐	☐	☐	☐	☐	☐	☐

Did you consume this sample with anyone (for e.g., talking with family/friend, etc.)?

☐ Yes

☐ No
